# Supplementary material for: Comparative Analysis and Phylogenetic Relationships of Ceriops Species (Rhizophoraceae) and Avicennia lanata (Acanthaceae): Insight into the Chloroplast Genome Evolution between Middle and Seaward Zones of Mangrove Forests
Source: Biology (Basel). 2022 Feb 28;11(3):383. doi: 10.3390/biology11030383 (PMC8945693; doi:10.3390/biology11030383)
Supplement: Supplementary file 1 [file FigureS1.pdf › FigureS1.pdf]

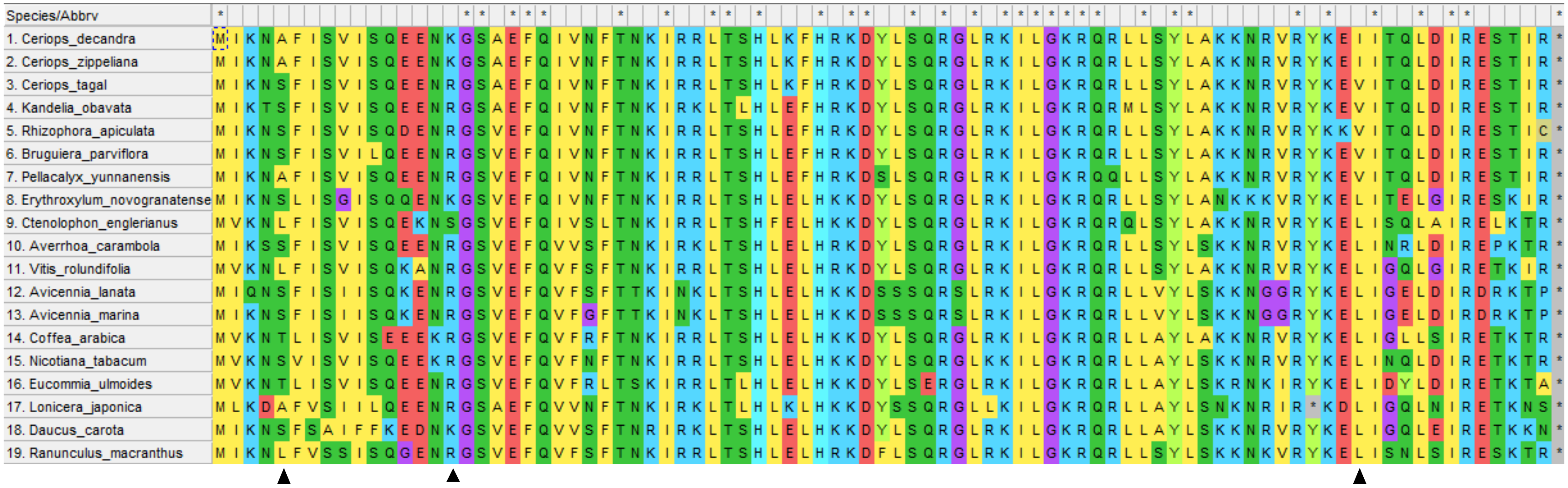

Figure S1. Multiple protein alignment of *rps15* genes among 19 species
